# Development of Folate-Group Impedimetric Biosensor Based on Polypyrrole Nanotubes Decorated with Gold Nanoparticles

**DOI:** 10.3390/bios12110970

**Published:** 2022-11-04

**Authors:** Andrei E. Deller, Ana L. Soares, Jaqueline Volpe, Jean G. A. Ruthes, Dênio E. P. Souto, Marcio Vidotti

**Affiliations:** 1 Grupo de Pesquisa em Macromoléculas e Interfaces, Universidade Federal do Paraná (UFPR), Curitiba 81531-980, PR, Brazil; 2 Laboratório de Espectrometria, Sensores e Biossensores, Universidade Federal do Paraná (UFPR), Curitiba 81531-980, PR, Brazil

**Keywords:** modified electrode, impedimetric biosensor, folate

## Abstract

In this study, polypyrrole nanotubes (PPy-NT) and gold nanoparticles (AuNPs) were electrochemically synthesized to form a hybrid material and used as an electroactive layer for the attachment of proteins for the construction of a high-performance biosensor. Besides the enhancement of intrinsic conductivity of the PPy-NT, the AuNPs act as an anchor group for the formation of self-assembly monolayers (SAMs) from the gold–sulfur covalent interaction between gold and Mercaptopropionic acid (MPA). This material was used to evaluate the viability and performance of the platform developed for biosensing, and three different biological approaches were tested: first, the Avidin-HRP/Biotin couple and characterizations were made by using cyclic voltammetry (CV) and electrochemical impedance spectroscopy (EIS), wherein we detected Biotin in a linear range of 100–900 fmol L^−1^. The studies continued with folate group biomolecules, using the folate receptor α (FR-α) as a bioreceptor. Tests with anti-FR antibody detection were performed, and the results obtained indicate a linear range of detection from 0.001 to 6.70 pmol L^−1^. The same FR-α receptor was used for Folic Acid detection, and the results showed a limit of detection of 0.030 nmol L^−1^ and a limit of quantification of 90 pmol L^−1^. The results indicate that the proposed biosensor is sensitive and capable of operating in a range of clinical interests.

## 1. Introduction

The development of electrochemical biosensors has been extensively explored so far. With these devices, the selective detection of low concentrations of different analytes, such as contaminants and biomolecules, is performed in a rapid and straightforward way; in addition, other features are highly desirable, such as low-cost, easy operation, portability, and no need of further analytical steps, as these are key parameters to obtaining an advantageous alternative to the traditional monitoring methods, which are often expensive and also not accessible to the entire population [1,2].

The construction of a high-performance electrochemical biosensor relies on a previous study on the material interface and transduction. Different assemblies of materials and architectures are possible in terms of nanomaterials, metals, and biomolecules to enhance both detection and quantification [3,4]. Special care must be taken on the biomolecule immobilization on the electrode surface, as this experimental step, consisting of the bioreceptor attachment needing to be stable, preserves its conformation and maintains a good orientation to interact with the analyte and provides a reliable signal of recognition [5].

Many different methodologies have been described along the past years [6,7,8], and in this context, the use of conducting polymers (CPs) and nanoparticles as hybrid synergist materials presents several advantages, not only for biosensors but for any electrochemical-based technology [9,10,11]. Among CPs, polypyrrole (PPy) plays an important role in electrode modification, as it can be further chemically prepared to attach biomolecules [7,12]. For biosensing, gold nanoparticles (AuNPs) are widely employed, as they present some interesting advantage based on biocompatibility, chemical affinity with sulfur ending molecules, besides the intrinsic metallic conductivity, which represents a rapid and reliable electrochemical transduction signal [13,14,15]. This last point is a key feature for the development of impedimetric biosensors which presents a remarkable sensitivity of detection; thus, it is possible to obtain trustable results in different stages, even early periods, of any disease [16,17]. Besides that, the impedimetric sensor proposed herein depends greatly on the better accuracy on the measure of the electric resistance of the transducer, so the higher the conductivity, the better will be the analytical parameters.

The folate group molecules have been found to possess different biological functions, such as cellular regulation, DNA synthesis, reparation, and methylation. It is important to adequately maintain the folate levels, as cardiovascular diseases, anemia, embryonic disorders, and various types of cancer are highly related to those levels [18,19,20]. Mammals do not synthetize folate, so its ingestion as vitamin B9 controls the adequate concentration in organisms [21]. The absorption of folate is performed by three different mediators: the reduced folate carrier (RFC); the proton-coupled folate transporter (PCFT); and the folate-binding proteins (FBPs), e.g., the folate receptor (FR-α) [22,23]. The interaction between FA and FR-α has a high specificity (KD = 10^−9^ mol L^−1^), so this strong interaction can be explored for the biosensor transduction mechanism. Recent studies indicate that the normal levels of FA in the human serum are around 11.3–34.0 nmol L^−1^, emphasizing the need for a highly sensitive biosensor [24,25]. In this study, we developed a hybrid nanomaterial formed by polypyrrole nanotubes and gold nanoparticles, electrochemically synthesized in a rapid and straightforward methodology. This modified electrode was employed as a platform to build up the well-known self-assembly monolayer (SAM) based on thiol chemical bonds and the attachment of biomolecules for further detection and quantification, using electrochemical impedance spectroscopy. All steps were properly characterized as well.

## 2. Materials and Methods

### 2.1. Reagents and Solutions

All solutions were prepared with ultrapure water (ElgaLab water 18 MΩ cm^−1^). Pyrrole (PI, Aldrich, San Luis, MO, USA) was distilled before use. Methyl orange (MO, Aldrich), nitric acid (HNO_3_, Synth), gold chloride trihydrate (III) (HAuCl_4_.3H_2_O, Aldrich), ethylenediaminetetraacetic acid (EDTA, Aldrich), and potassium chloride (KCl, Aldrich) were used as received, without any further purification step. Mercaptopropionic acid (MPA, Aldrich), N-ethyl-N-(3-dimethylaminopropyl) carbodiimide (EDC, Aldrich), N-hydroxysuccinimide (NHS, Aldrich), and amino acetic acid (Glycine, Aldrich) were kept in a refrigerator at 5 °C. The biological samples, Avidin/Biotin couplings, avidin conjugated with horseradish peroxidase (Avidin-HRP, Abcam, Cambridge, UK), anti-avidin antibody (Biotin, Abcam), recombinant human folate binding protein (FBP, Abcam), and anti-folate binding protein antibody (FBP-Ab, Abcam), were kept in a refrigerator at 5 °C.

### 2.2. Characterization and Electrochemical Measurements

For the electrochemical experiments, Metrohm DropSens μStat-i 400s potentiostat was employed. The EIS and CV were performed in PBS buffer 0.1 mol L^−1^ at pH 7.4; as the reference electrode, we used Ag/AgCl/KCl_sat_, and platinum wire served as a counter electrode. The working electrode was 316 steel mesh–400 mesh, previously cleaned by immersion in ethanol and ultrapure water. The spectroscopic and microscopic characterizations were performed in UFPR Electronic Microscopy Center (CME-UFPR), with Tescan Vega3 LMU equipment and Transmission Electron Microscopy (MET) with JEOL JEM 1200EX-II equipment with 0.5 nm resolution. All experiments were performed in triplicate to assure homogeneity and reliability of the results.

### 2.3. Electrode Preparation and Electrochemical Synthesis of PPy/AuNPs

The electrochemical synthesis of PPy nanotubes was performed in aqueous solution containing 100 mmol L^−1^ of pyrrole monomer, methyl orange (MO) 5 mmol L^−1^, and 8 mmol L^−1^ KNO_3_; the pH 3 was adjusted by dropping HNO_3_ solution. The electrochemical synthesis was performed over the steel mesh by potentiostatic method, applying 0.8 V over time, controlling the amount of polymer over the mesh with charge control of 0.5 C cm^−2^ [26].

The AuNPs deposition into PPy was performed in a solution of 1.0 mmol L^−1^ HAuCl_4_, 0.17 mol L^−1^ K_2_HPO_4_, 0.036 mol L^−1^ Na_2_SO_3_, and 0.48 mmol L^−1^ EDTA. The chemicals were added in this sequence to avoid the darkening of the solution, due to gold precipitation. The electrodeposition was performed by chronoamperometry, applying −1.1 V vs. Ag/AgCl/Cl_-sat_, with charge control of 300 mC cm^−2^ [27,28].

### 2.4. Biosensor Construction and Characterization

For biosensor construction, the formation of a favorable environment for the biomolecule immobilization is necessary. Gold has a strong interaction with sulfur, so organic molecules with thiol groups can be easily anchored onto the AuNPs surface by stable covalent bonds [29]. This affinity and stability are explored in SAMs formation, producing an organized and compatible electrode surface for the immobilization of biomolecules.

**Figure 1:**
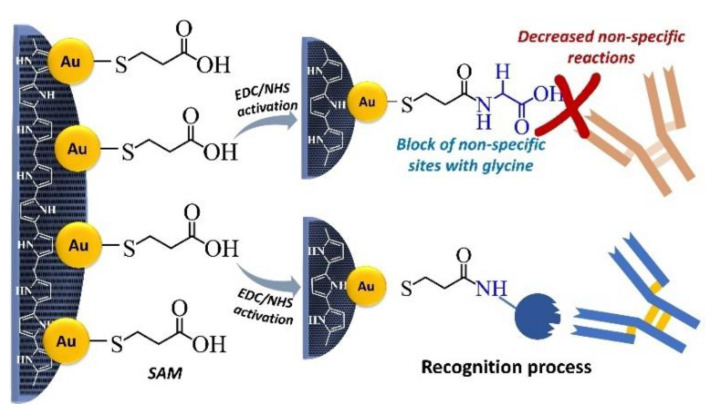
The SAM formation is due to the covalent interaction between gold and sulfur, which makes possible biomolecule immobilization through the carboxylic groups.

The methodology for biosensor construction was the same for all the biological systems studied. The modified electrode (PPy/AuNPs) was immersed into MPA 1 mmol L^−1^ aqueous solution for five hours to SAM formation and then was washed in ultrapure water for 15 min. Thus was followed by activation with 100 and 150 mmol L^−1^ EDC:NHS aqueous solution for 20 min. Then it was washed in ultrapure water for 1 min. After activation, the biorecognition element was immobilized by immerging the electrode in a solution of the respective biomolecule for 45 min, followed by a cleansing step in PBS for 15 min. For the complex Avidin/Biotin, both were tested as a bioreceptor in the concentration of 25 µg mL^−1^. Moreover, in the other two tests evaluated for the folate biomarker, the same bioreceptor was explored: FBP 8 nmol L^−1^. The next step was blocking unspecific active sites with glycine 100 mmol L^−1^ by submerging the electrode into the glycine solution for 15 min. In Figure 1, the basic steps of the SAM formation are shown.

The detection of the biomolecule analyte followed the same methodology, where the electrode was immersed in a solution containing the analyte at a known concentration, followed by a washing step in PBS for 5 min before CV and EIS measurements [28,30]. The impedimetric results were modeled by using the proper equivalent circuit and values obtained from NOVA software.

## 3. Results

### 3.1. Electrode Modification and Characterizations

**Figure 2:**
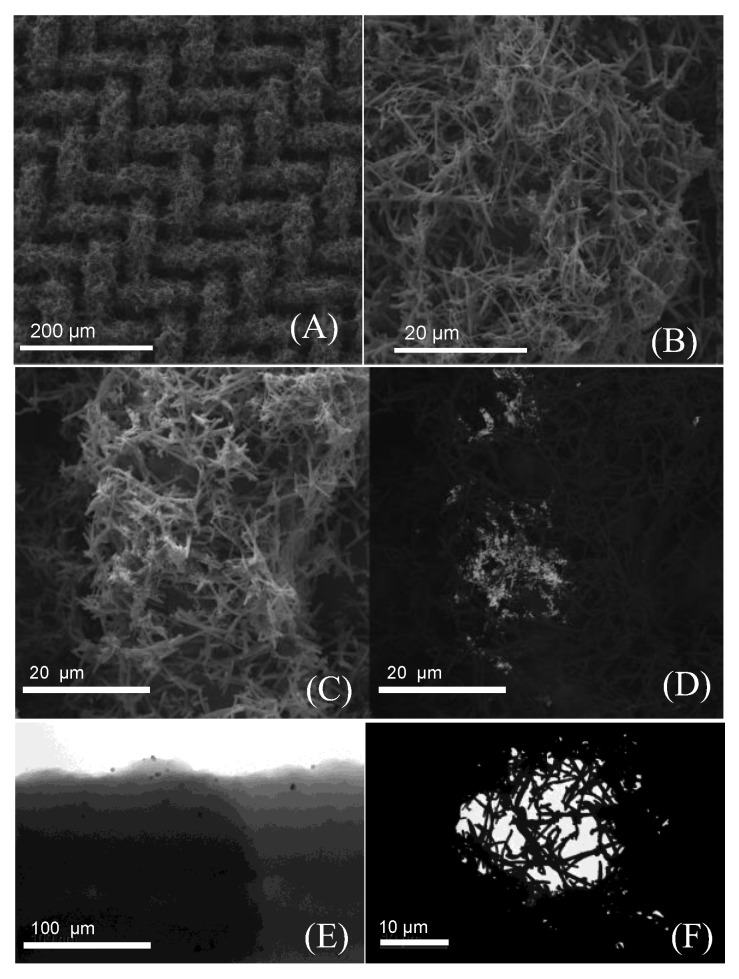
Representative SEM images from the steel mesh coverage: (**A**,**B**) closer approximation of a wire mesh, (**C**) the wire-mesh image of secondary electrons of the hybrid PPy/AuNPs, and (**D**) the SEM with backscattered electrons. (**E**,**F**) TEM representative images from a single nanotube and a small gap in between the steel mash, respectively.

**Figure A1:**
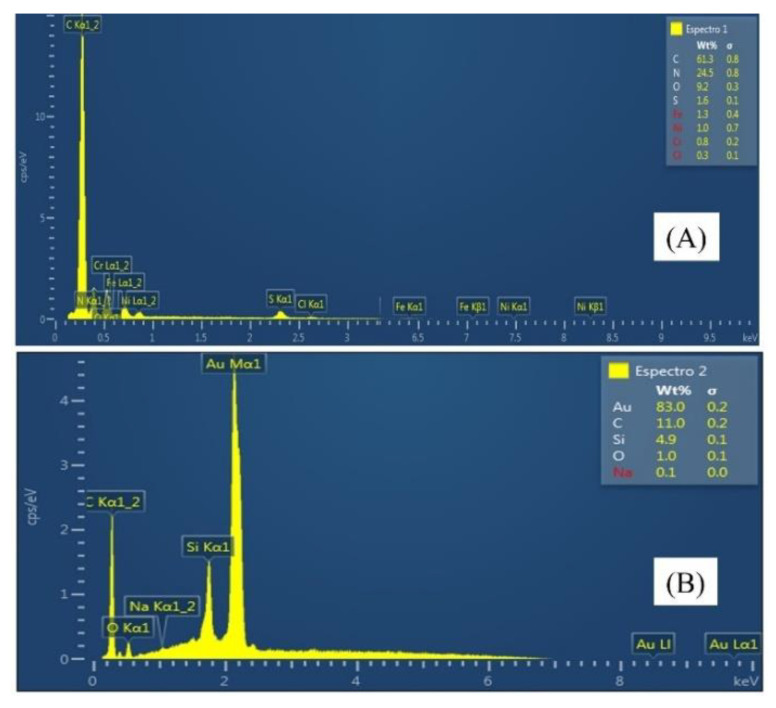
EDS spectra were obtained for the characterization of the electrode modified with PPy (**A**) and the electrode modified with PPy/AuNPs (**B**).

The PPy-NT/AuNPs-modified electrodes were characterized by TEM and SEM, as shown in Figure 2. The nanotube morphology is clearly present and fully covered the mesh substrate (Figure 2A,B). The AuNPs can be seen in Figure 2C and in more detail in Figure 2D, using backscattered electron images (Figure 2D); the gold presence was also corroborated by EDS spectrum (Figure A1). The TEM images show individual AuNPs (Figure 2E) with very few nanometers spread along the PPy-NT’s surface. Using TEM, it was also possible to verify the filling of the mesh structure with the polymer nanotubes (Figure 2F).

The electrochemical characterization of modified electrodes relies on two fundamental techniques, cyclic voltammetry (CV) and electrochemical impedance spectroscopy (EIS). These two must be studied in consonance to obtain valuable information about the electrode kinetics, adsorption and fouling effects, electron transfer, mass transport effects, steady state conditions, and so on. For EIS studies, it is important to adopt an equivalent circuit model to better understand and quantify different processes at the electrode surface; to date, the Randles modified circuit is very common in the study of conductive-polymer-modified electrodes [31,32]. For the biosensor proposed herein, the main information obtained by the EIS technique is associated with the biomolecule interaction, such as antigen–antibody, a so-called affinity interaction caused by the changes at the interface of the electrochemical active material, in terms of both charge transfer and double-layer effects [1,5,31].

**Figure 3:**
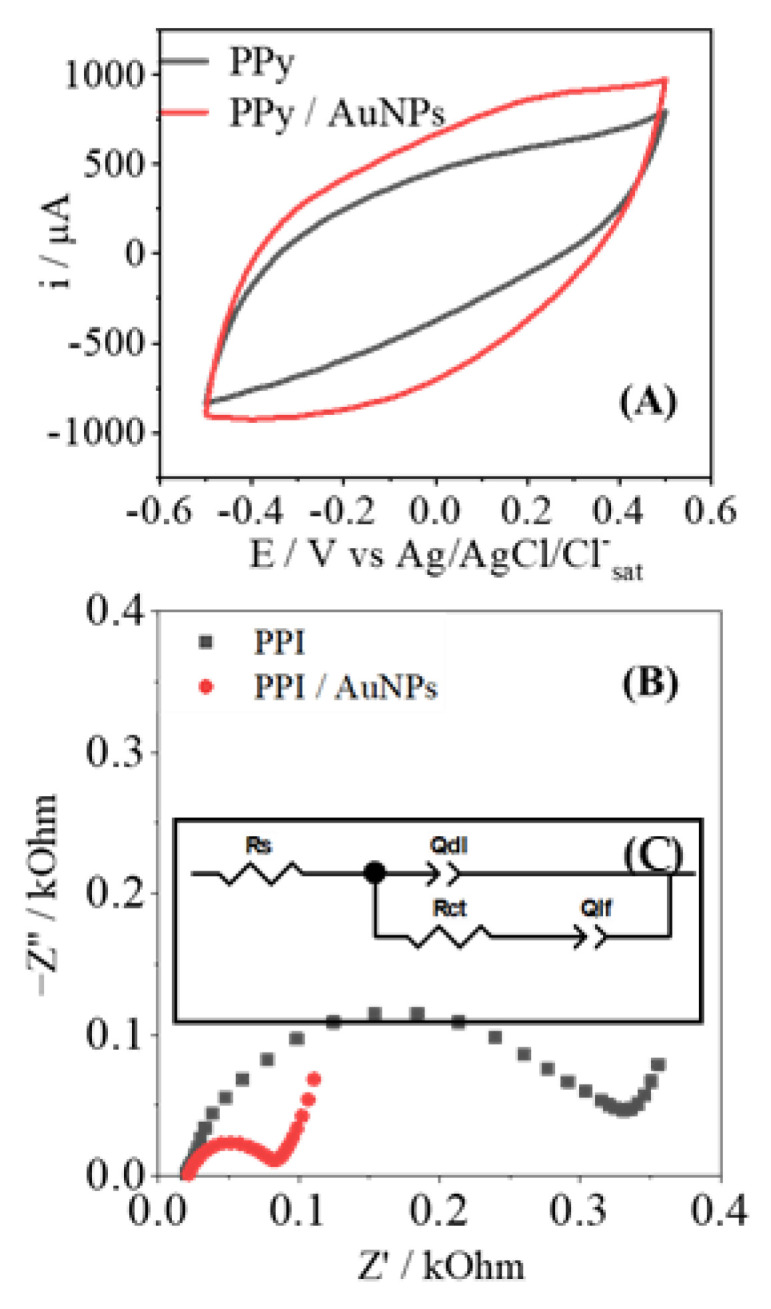
(**A**) CV for the electrodes modified with just PPy (black) and PPy/AuNPs (red). The Nyquist plot is shown in (**B**) from electrodes modified with PPy (black) and with PPy/AuNPs (red). The equivalent circuit used to model the EIS results is also inserted as (**C**).

Electrochemical experiments of CV and EIS were performed to characterize and compare the proprieties between PPy-NTs- and PPy-NTs/AuNPs-modified electrodes. Figure 3A shows the CVs for each modified electrode, and it is possible to observe an increment in the current in the presence of AuNPs. It is important to note that no additional redox processes are observed; there is solely an increment of the capacitive current, indicating an increase of the electroactive surface provoked by the exposure of a large area of the AuNPs. Figure 3B shows the Nyquist plots of the modified electrodes; they show a traditional semicircle response that is characteristic of conducting polymers. Clearly there is a drastic decrease in the semicircle radius in the presence of AuNPs; in general lines, this behavior indicates an increase in the electroactivity of the interface, thus corroborating the presence of a metallic structure on a polymeric matrix. The equivalent circuit used to fit the electrochemical parameters is found in Figure 3C; they can be summarized as follows: the Q_DL_ parameter is related to the energy of the double layer at the interface electrode/electrolyte, the R_CT_ is the resistance of the charge transfer at the electrode surface, R_S_ is the resistance of the solution, and Q_LF_ deals with the number of interacted ions inserted within the polymeric matrix.

The experimental results obtained in Figure 3B were modeled according to the equivalent circuit shown in Figure 3C; the results are shown in Table 1. As discussed previously, there is a significant improvement in the charge transfer in the polymer/electrode interface with the AuNPs, as indicated by the lower value of R_CT_. It is important to add that the presence of a metallic particle itself contributes to the increment of conductivity of the PPy-NTs, and this also facilitates any electron transfer at the surface. Due to the high superficial area of AuNPs, the Q_DL_ value shows an increment of almost 2.5 times, in agreement with the increase that the capacitive current showed in CV. At a low frequency, the Q_LF_ value had no significant variations, indicating that the intercalation of charges in the polymeric matrix is not affected by the presence of AuNPs; this seems reasonable, as the amount of polymer was kept the same, at the same cutoff charge. Regarding the morphology, after the AuNPs’ deposition, it was possible to observe a decrease in the n_DL_ and n_LF_ parameters, which represent the escape from ideality of a traditional parallel capacitor, which represents *n* = 1; thus, the further away it is from the unity, the rougher the surface is present at the electrode surface [33,34].

### 3.2. Functionalized Steel Mesh Electrode (PPy/AuNPs/MPA) for Biosensing Applications

#### Avidin-HRP/Biotin Complex: A Model System

**Figure A2:**
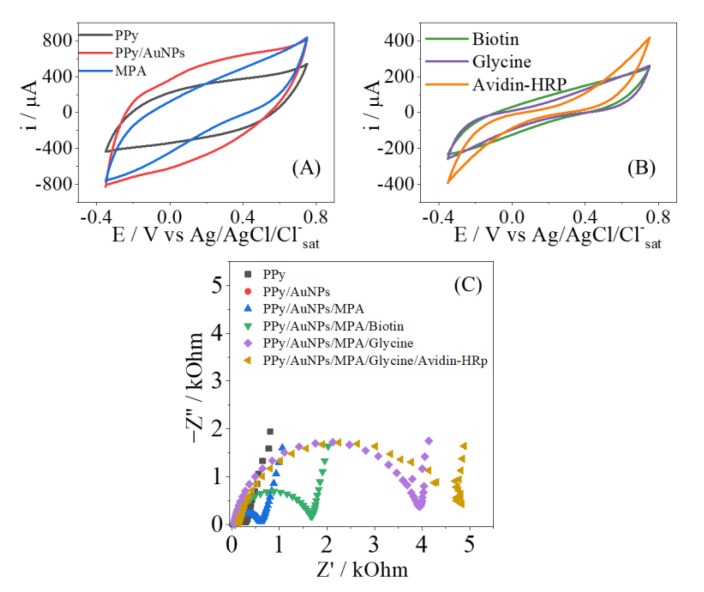
Characterization of the PPy/AuNPs-based biosensor construction steps for the Avidin-HRP/Biotin couple. Cyclic voltammograms (**A**,**B**) show the characterization of different steps in the construction of the platform-based biosensor, and the Nyquist diagram (**C**) is for the same characterization.

The steps of the biosensor construction were characterized electrochemically by CV and EIS, as shown in Appendix A Figure A1 and Figure A2, where the blocking of the surface can be easily identified. The availability for the attachment of biomolecules was performed by the Avidin-HRP protein to detect Biotin, as a well-known system, possessing a very strong interaction. Avidin is a basic tetrameric glycoprotein composed of four identical subunits, and each of these subunits can bind to Biotin with high stability and affinity, being one of nature’s strongest non-covalent interactions (dissociation constant = 10^−15^ mol L^−1^). Thus, this interaction can be used to verify the effectiveness of the modified electrode, as shown elsewhere [35,36].

**Figure 4:**
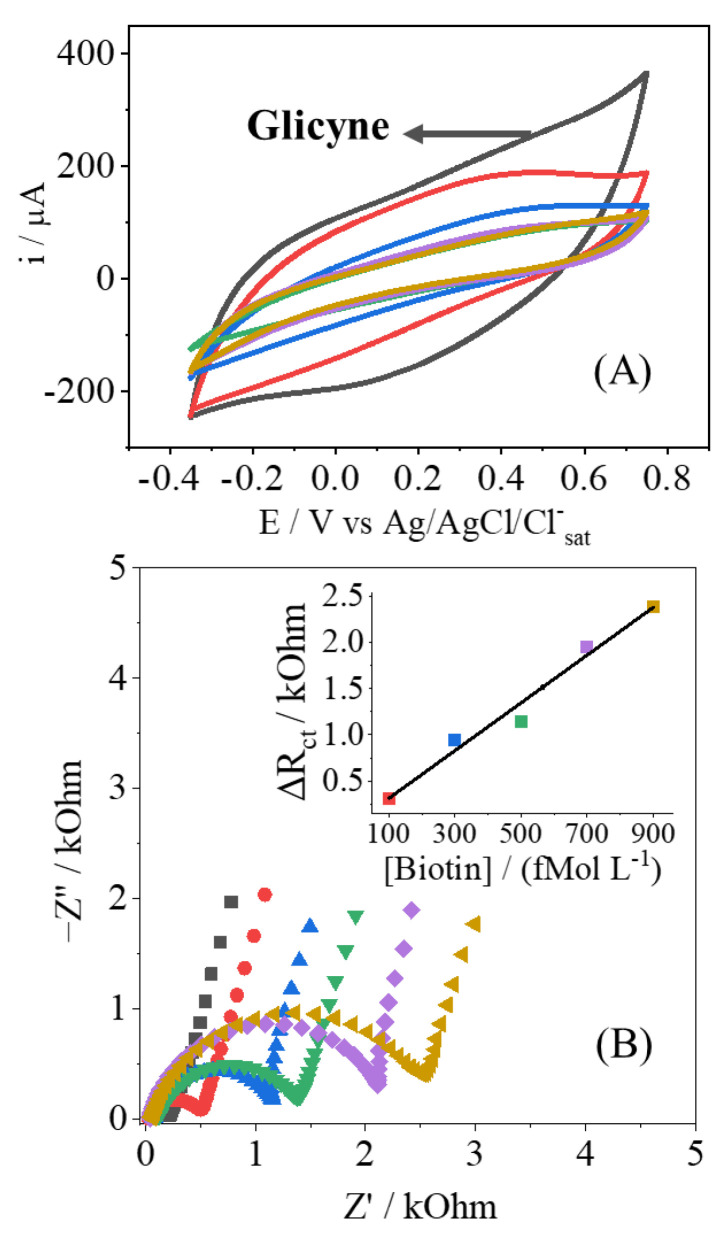
Cyclic voltammetry (**A**) and Nyquist plot (**B**) of the EIS measurement to Biotin detection (100 up to 900 fmol L^−1^) indicated by colors in both CV and EIS.

In Figure 4A, it is shown how the concentration of Biotin affects the voltammetric response of the electrode. The voltammogram just after the blocking of glycine is shown for the sake of comparison, as no Biotin is added. Clearly the CVs present a diminishment of the current response, indicating the adsorption of Biotin at the electrode surface, where some active sites are no longer available. This effect is also observed in the Nyquist plots (Figure 4B), with the change of the R_CT_ parameter, as observed in other contributions [28,37]. As the concentration of the insulating Biotin increases, more electroactive sites are being hindering, so there is the increment of the resistance of any potential redox reaction; since this behavior is related to the amount of analyte, a proper analytical curve can be drawn, as shown. The EIS results of Figure 4B were modeled, as mentioned before, and the results are shown in Table 2. Besides the variation of the R_CT_, the Q_DL_ parameter also changes, indicating that the double layer is also affected by the presence of Biotin, corroborating the strong adsorption at the electrode’s surface. The other parameters have shown no drastic changes, and this outcome is in consonance with no redox reactions promoted by the PPy-NT electrodes.

These results obtained with the avidin/biotin biological system indicate the interesting behavior of PPy-NTs/AuNPs-modified electrodes for the construction of biosensors based on electrochemical response, as is later discussed.

### 3.3. Biosensor for Folate Detection from the Disposable Electrode Modified by PPy/AuNPs/MPA

#### 3.3.1. Biofunctionalization Step: Recombinant Human Folate Binding Protein (FBP, Abcam) as Recognition Element

**Figure 5:**
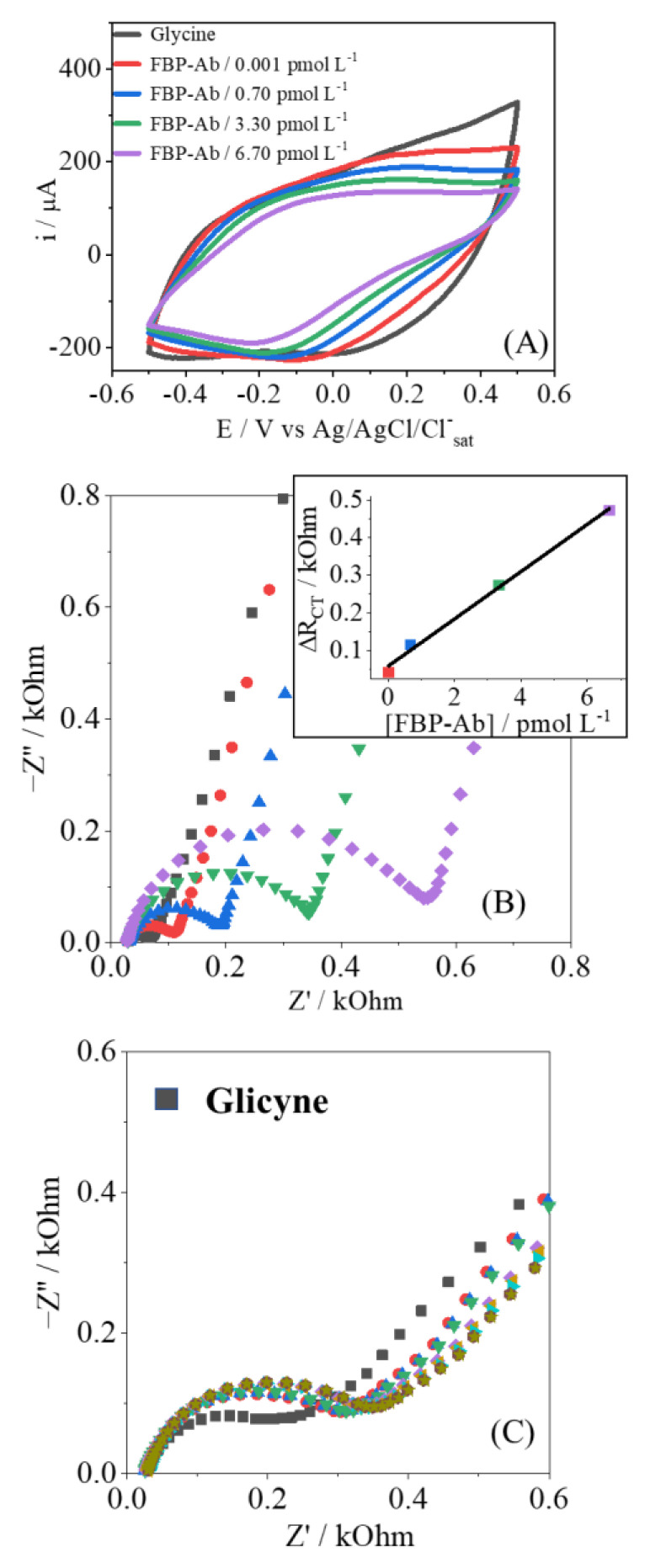
Cyclic voltammetry (**A**) and Nyquist plot (**B**) to FBP-Ab detection (0.001 up to 6.70 pmol L^−1^); (**C**) the EIS response in stability test to 0.001 pmol L^−1^ of FBP-Ab. The gray measurement was performed in the blank step, while the others correspond to the same antibody concentration.

After the interesting results presented by the PPy-NTs/AuNPs electrodes for the Avidin/Biotin biomolecules, the same platform was used for the construction of FBP-Ab/FBP biosensor. In the same perspective observed in Figure 4, the CV and EIS responses in the presence of FBP-Ab are shown in Figure 5, and a similar behavior was found, indicating that the same effects of strong interaction and adsorption are occurring.

To test the stability of the recognition process, several measurements of EIS were performed for the same antibody concentration, as shown in Figure 5C and Table A1 and Table A2. After immersion in FBP-Ab, five measurements in a row were performed, applying analysis of variance (ANOVA) with 95% confidence. The R_CT_ parameter showed no significant difference, maintaining the confidence in the analytical response; this point is related to the strong interaction between the biosensor and analyte, with no desorption of the FBP-Ab from the electrode’s surface [38].

**Figure A3:**
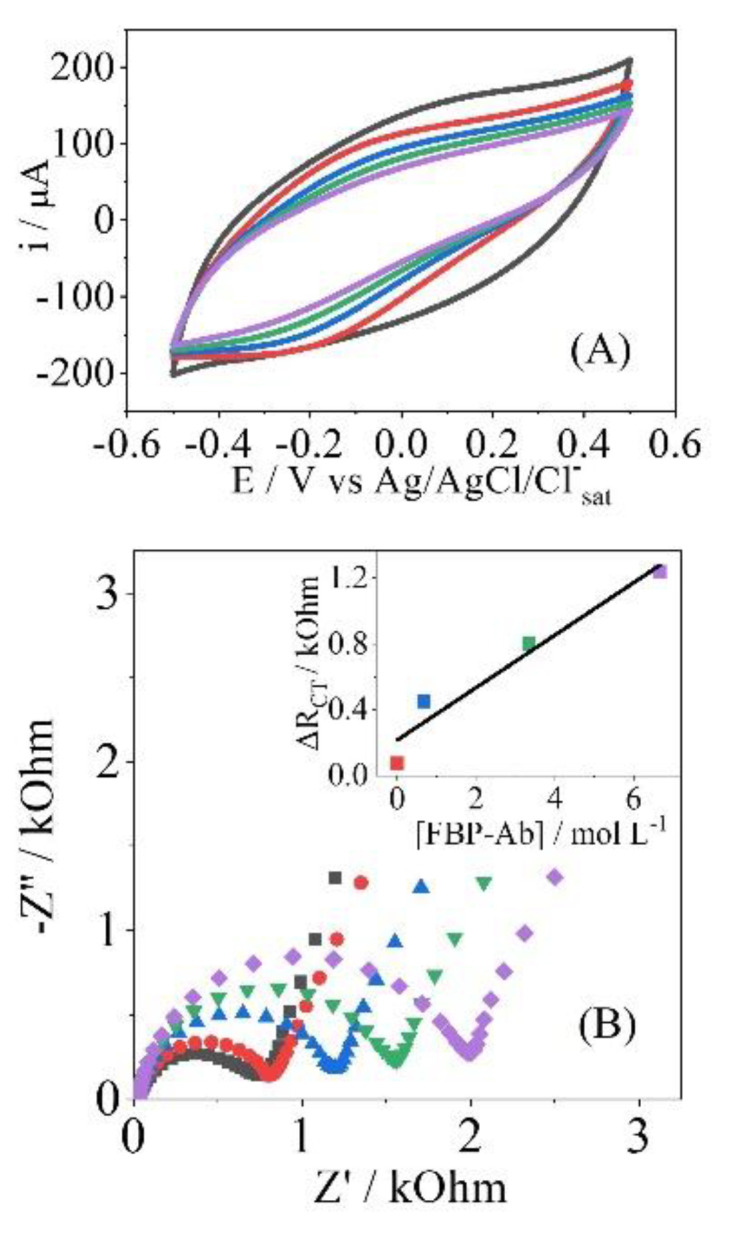
Adsorption test of different antibody concentrations (0.001, 0.67, 3.30, and 6.60 pmol L^−1^), using a modified electrode only with PPy. Cyclic voltammograms (**A**), Nyquist diagram (**B**).

We also tested and proved that the glycine blocking step is crucial. It is already known that the adsorption of biomolecules in conductive polymers can cause non-specific interactions on the electrode’s surface, interfering with the signal [39]. We performed a test shown in Appendix A Figure A3, where we verified that, without a blocking step, it is possible to have nonspecific antibody adsorption on the polymer matrix, which directly interferes with the signal.

#### 3.3.2. Detection Step: Determination of Femtomolar Concentrations of Folic Acid

**Figure 6:**
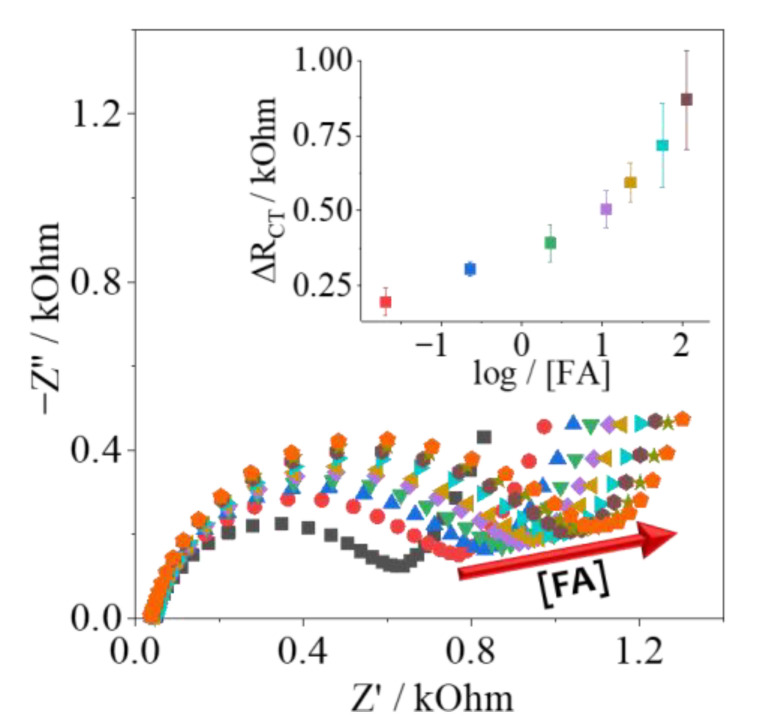
Folic Acid detection (0.02 up to 113.3 nmol L^−1^) using the PPy/AuNPs-modified electrode.

Finally, the FBP/Folic Acid biosensor was assembled on the PPy-NT/AuNPs platform, all electrochemical experiments were the same ones descried earlier for the detection of the analyte. Folic Acid has a great affinity for FBP, and the impedimetric response is found in Figure 6, in the concentration range from 0.02 up to 113.3 nmol L^−1^, in triplicate. The analytical curve was inserted; the limit of detection (LOD) was calculated as 0.030 nmol L^−1^, and the limit of quantification (LOQ) was 0.090 nmol L^−1^, indicating that the proposed biosensor herein can detect and quantify the range of concentration of clinical interest, which is around 11 up to 34 nmol L^−1^ [24,25]. As this biomarker can be found as a group of molecules, many different configurations of biosensors based on folate can be found in the literature, and the simple comparison between analytical parameters is not always easy to study. Nonetheless, in Table 3, different information is presented to better analyze the recent development in this issue.

## 4. Conclusions

The electrode modification with PPy-NTs/AuNPs has shown to be rapid, straightforward, and reliable for the construction of biosensors. This hybrid material was used as a platform for SAM layers, followed by the anchoring of different biomolecules, indicating a potential application in different types of biosensors and recognition elements. All characterization experiments corroborated the influence of the nanometric architecture on the electrochemical response for the detection and quantification of different analytes, with the R_CT_ parameter showing the most sensible response for the biological recognition of the biological markers. The nanostructures also are responsible for the possibility of detection in the range of femtomolar to picomolar, corroborating the great sensitivity achieved by the combination of the nanostructures, specific adsorption, and impedance technique.

## Figures and Tables

**Table 1:** Parameters’ values obtained by EIS to PPy e PPy/AuNPs, obtained from fitting of EIS results, R^2^ > 0.99. The equivalent circuit was modeled by the NOVA software.

	R_S_/kΩ	Q_DL_ 10^−5^ F s^n−1^	n_DL_	R_CT_/kΩ	Q_LF_ 10^−3^ F s^n−1^	n_LF_
PPy-NTs	0.04	1.89	0.82	0.25	3.60	0.83
PPy-NTs/AuNPs	0.03	4.27	0.76	0.05	6.70	0.77

**Table 2:** Parameters’ values obtained by EIS to PPy e PPy/AuNPs after fitting, R^2^ > 0.98.

	Glycine	Biotin Concentration (fmol L^−1^)				
		**100**	**300**	**500**	**700**	**900**
R_S_/kΩ	0.05	0.03	0.06	0.12	0.04	0.06
Q_DL_/10 ^−5^F s^n−1^	2.36	2.96	2.63	3.06	2.37	3.38
n_DL_	0.87	0.84	0.85	0.80	0.86	0.81
R_CT_/kΩ	0.16	0.48	1.11	1.31	2.11	2.56
Q_LF_/10^−3^ F s^n−1^	4.6	4.6	5.35	4.37	5.17	4.83
n_LF_	0.80	0.81	0.90	0.84	0.91	0.86

**Table 3:** Comparison between experimental conditions and LOD values between different biosensors for FA detection.

Material	Detection Method	Concentration Range (nmol L^−1^)	LOD (nmol L^−1^)	Reference
Steel mesh covered by PPy/AuNPs	EIS	0.02–113.3	0.030	This work
Gold/PPy/POM	Cyclic voltammetry	0.01–1	0.0075	[40]
Gold electrode modified with SAM	Square wave voltammetry	0.008–1	0.004	[41]
Hydroxyapatite NPs/GCE	Differential pulse voltammetry	0.1–350	0.075	[42]
Platinum NPs/MWCNT/GCE	Linear voltammetry	0.2–100	0.05	[43]
MoS_2_/rGO/GCE	Differential pulse voltammetry	0.1–100	0.01	[44]
Boron doped diamond electrode	Stripping voltammetry	0.23–45	0.08	[45]
PPy-modified sol–gel carbon ceramic	Differential pulse voltammetry	7–55	1.8	[46]
Chromatographic column	HPLC/UV–Vis	0.3–100	44.14	[42]
SPCE/GO	Amperometry	100–1.6 × 10^6^	20	[43]
SPCE/SWCNT	Square wave voltammetry	70–500 × 10^3^	800	[46]

## Appendix A

**Table A1:** Parameters’ values obtained by EIS to PPy e PPy/AuNPs, in electrode modification steps.

Parameter	MPA	Biotin	Glycine	Avidin-HRP
R_S_/kΩ	0.05	0.07	0.05	0.14
Q_DL_/10^−5^F s^n−1^	1.87	1.88	1.61	2.43
n_DL_	0.90	0.90	0.91	0.85
R_CT_/kΩ	0.56	1.62	3.95	4.67
Q_LF_/10^−3^ F s^n−1^	5.80	6.70	8.06	8.99
n_LF_	0.81	0.87	0.96	0.97

**Table A2:** Parameters’ values obtained by EIS to PPy e PPy/AuNPs for stabilization tests using 0.001 pmol L^−1^.

	Glycine	EIS Measurements to FBP-Ab to 0.001 pmol L^−1^							
R_CT_ (Ohm)	206.3	283.3	288.2	291.2	312.7	309.3	312.3	315.8	318.2
